# *Euterpe oleracea* extract inhibits tumorigenesis effect of the chemical carcinogen DMBA in breast experimental cancer

**DOI:** 10.1186/s12906-018-2183-z

**Published:** 2018-04-02

**Authors:** Jéssica Alessandra-Perini, Jamila Alessandra Perini, Karina Cristina Rodrigues-Baptista, Roberto Soares de Moura, Antonio Palumbo Junior, Thiago Alves dos Santos, Pergentino José Cunha Souza, Luiz Eurico Nasciutti, Daniel Escorsim Machado

**Affiliations:** 1 Morphological Sciences Program, Biomedical Sciences Institute, Federal University of Rio de Janeiro, Rio de Janeiro, RJ Brazil; 2 Research Laboratory of Pharmaceutical Sciences, West Zone State University, Av Manuel Caldeira de Alvarenga, 1.203, Campo Grande, Rio de Janeiro, RJ 23070-200 Brazil; 3 Program of Post-graduation in Public Health and Environment, National School of Public Health, Oswald Cruz Foundation, Rio de Janeiro, RJ Brazil; 4 Department of Pharmacology and Psychobiology, State University of Rio de Janeiro, Rio de Janeiro, RJ Brazil; 5 Departament of Pharmacy, Federal University of Pará, Belém, PA Brazil

**Keywords:** Breast cancer, *Euterpe oleracea*, Anti-inflammatory, Angiogenesis

## Abstract

**Background:**

Among the processes involved in the breast tumor microenvironment, angiogenesis and inflammation play a central role, and the main factors of these processes are the vascular endothelial growth factor (VEGF), cyclooxygenase 2 (COX-2) and macrophages. Recently, the extract of *Euterpe oleracea* (açaí), a fruit that is widely found in the Amazon region, already showed antitumorigenic effects in vitro in human breast cancer cell lines. The present study aimed to investigate the effect of açaí on breast cancer using a chemically DMBA (7,12-dimethylbenzanthracene) experimental model.

**Methods:**

One day after initiation of treatment with açaí, mammary carcinogenesis was induced in female Wistar rats using a subcutaneous injection of 25 mg/kg of DMBA in the mammary gland. Forty rats were randomized into two groups: treated with 200 mg/kg of either açaí extract or vehicle, via gastric tube for 16 consecutive weeks. After treatment, the tumor was collected for macroscopic, histological and immunohistochemical (VEGF, vascular endothelial growth factor receptor 2 -VEGFR-2, COX-2 and matrix metalloproteinase -MMP-9) analyses; peritoneal fluid was subjected to flow cytometry (F4–80/MAC-2+) and ELISA immunoassay (VEGF, prostaglandin E_2_ -PGE_2_ and interleukin-10 -IL-10). Heart, liver and kidney samples were collected for histological analysis.

**Results:**

After 16 weeks of induction, the mammary carcinoma was confirmed by macroscopic and histological evaluation. Survival analysis indicates that açaí increased the survival (*P* = .0002, long-rank test) and reduced the deaths number (*P* = .0036, Chi-square test). Açaí treatment decreased the number of inflammatory cells and macrophage positive cells (Mac-2 + F4–80+), as well as promoting a reduction in immunostaining of VEGF, VEGFR-2 and COX-2. The açaí group also exhibited lower concentrations of PGE_2_, VEGF and IL-10 compared to the control. The histopathological results of the liver and kidneys showed protective effect of açaí, since in the control group, there was an increase in fibrosis, atypical cells and hemorrhagic microenvironment.

**Conclusion:**

The results of this study demonstrated the antiangiogenic and anti-inflammatory potential of açaí, like due to the decreases of the number of activated macrophages, resulting in the inhibition of DMBA carcinogenicity in breast cancer.

**Electronic supplementary material:**

The online version of this article (10.1186/s12906-018-2183-z) contains supplementary material, which is available to authorized users.

## Background

Breast cancer is the most frequently diagnosed cancer in women worldwide, excluding non-melanoma skin tumors [1, 2]. In the current year, 252,710 new cases of breast cancer are expected, which corresponds to 30% of all female cancer cases that will be diagnosed in 2017 [1, 2]. Furthermore, breast cancer represents the second most common cause of cancer-related death in women worldwide. This year, 40,610 tumor-related deaths are expected, which represents 14% of all cancer-related deaths in women [1, 2]. This scenario is also observed in Brazil; in 2017, 57,960 new cases of breast cancer are expected (28. 1% of all female cancer cases), which makes breast cancer the most common malignancy to afflict Brazilian women [3]. Therefore, breast cancer presents a social impact that cannot be neglected, since approximately 15 thousand deaths are caused by this cancer in Brazil each year [4].

The exact process by which breast cancer is initiated is yet to be elucidated, but in the past decade, several studies have demonstrated that angiogenesis and inflammation are essential for the growth, invasion and metastasis of primary tumors, including breast cancer [5–7]. In this sense, among the several angiogenic factors previously described, the Vascular Endothelial Growth Factor (VEGF) represents the main molecule in the angiogenesis process because it mediates key events in the formation of new vessels [8]. In fact, clinical studies have been shown that high levels of VEGF in breast cancer patients are associated with a poor prognosis, tumor recurrence and a decrease in the overall survival [9–11]. On the other hand, the role of the inflammatory mediator Cyclooxygenase 2 (COX-2) has been reported as a key player in the tumorigenesis of several tumor types, including breast cancer [12–14]. Furthermore, COX-2 has been also associated with worse prognosis [13–15], tumor growth and metastasis [14, 15].

The current management for breast cancer, includes surgery, chemo and radiotherapy, besides hormone and anti-Her-2 therapies [16, 17]. Despite the vast options of treatment and the advancements in breast cancer therapy, the therapeutic approach currently used in treatment of disease still produces many side effects, which directly impact in the quality of life and the success of the treatment [17, 18]. Therefore, the search for therapies that could reduce the side effects, alone or in combination with drugs already used in the treatment of breast cancer, is an essential step to increase the disease management.

*Euterpe oleracea* Mart. (Arecaceae), commonly known as “açaí”, is a palm fruit native from the Amazon region of Brazil [19] whose antioxidant, antinociceptive, anti-inflammatory, and anticancer activities have been previously reported as “natural” therapeutic options in the treatment of several pathologic conditions [20-28]. In fact, recent data from Silva and colleagues showed that açaí extract exerts an antitumorigenic effect in breast cancer malignant cells by inducing an increase in the autophagy process, in addition to decreasing the cellular viability of MCF-7 cells [26]. In addition, our group previously demonstrated that açaí plays a remarkable antiangiogenic and anti-inflammatory role in endometriosis, which is a benign disease that presents a largely known malignant behavior [28]. Furthermore, Ribeiro et al. [29] demonstrated that the genotoxic effects of doxorubicin treatment was attenuated by the acute and subacute açaí treatment in mice due to a decrease in the cardiotoxicity promoted by doxorubicin chemotherapeutic agent.

Due the promising therapeutic potential of açaí, in the present study, we investigated the effects of açaí extract on the establishment and growth of breast tumors in a chemically experimental model using the DMBA, as well its role in the angiogenesis and inflammatory process.

## Methods

### Preparation of the extract from açaí

*Euterpe oleracea* Mart. fruits were obtained from the Amazon Bay (Belém do Pará, Pará, Brazil), and identified by curator Ricardo de S. Secco, Herbarium Museum Paraense Emílio Goeldi (Belém do Pará, Pará, Brazil). The plant specimen was deposited in the same herbarium with the voucher specimen MG 205222 number. The hydroalcoholic solution extracted from açaí stones was prepared as previously described [22, 24, 27, 28]. In summary, 200 g of açaí stone were boiled in 400 mL of distilled water for 10 min and mixed for 2 min. The decoction was allowed to cool at room temperature and extracted with 400 mL of ethanol shaking for 2 h. The extract was kept at 4 °C for 10 days and filtered through Whatman filter paper and the ethanol was evaporated (Fisatom Equipamentos Científicos Ltda São Paulo, São Paulo, Brazil) under low pressure at 55 °C. Then the extract was lyophilized (Fisatom Equipamentos Científicos Ltda São Paulo) at temperatures from − 30 to − 40 °C and under a vacuum of 200 mmHg, and frozen at − 20 °C until use.

### Breast cancer experimental model

The Institutional Animal Care and Use Committee (CEA) of West Zone State University (UEZO) approved the protocols used in this study (protocol code CEA-UEZO-008/2014). All experiments were conducted in accordance with the Ethical Guidelines from the CEA and the NIH Guidelines for the Care and Use of Laboratory Animals (http://oacu.od.nih.gov/regs/index.htm. 8th Edition; 2011).

Experiments were carried out with 8-week-old female Wistar rats weighing about 150–200 g. The mice were housed in polyethylene cages in the Bioterium of UEZO, and were kept in a room with a constant temperature (25 °C) under a 12-h light/dark cycle with free access to food and water.

Using the method described by Deepalakshmi and Mirunalini [30] and Cerqueira-Coutinho et al. [31], the breast tumor was induced by a single subcutaneous injection in the mammary region of 25 mg of 7,12-dimethylbenzanthracene (DMBA) in 0,5 mL of sunflower oil and 0,5 mL of physiological saline. The DMBA was used as per the care manufacturer’s instruction.

### Açaí treatment

One day before the tumor induction, the 40 rats were divided randomly into two groups of each twenty animals: the açaí group was treated with 200 mg/kg body weight [28, 32, 33], dissolved in saline, and the control group received saline as vehicle. Both groups were administered daily by gastric tube for 16 consecutive weeks. Animals were palpated in the mammary gland once a week to detect the presence of breast tumors. At the end of the 16-week treatment period, the animals were euthanized by anesthesia overdose (ketamine and xylazine), and the peritoneal fluid was collected for flow cytometry and ELISA immunoassay. Tumor tissue, heart, liver and kidneys were collected and fixed in 10% buffered formalin and embedded in paraffin for histological analyses; tumor tissue was also used for the immunohistochemical studies. All macroscopic mammary tumors were counted, excised, and weighed. The tumor volumes was measured (length x width) to the nearest 0.1 mm, using calipers and was calculated according to the following formula: Tumor volume = 1/2 (length x width^2^).

### Histology, immunohistochemistry and morphometric analysis

Formalin-fixed mammary glands were paraffin-embedded and cut into 4-μm-thick sections. Part of the sections were stained with Harris hematoxylin and eosin (HE), and examined microscopically at 200× magnification for the presence of histological hallmarks of breast cancer. The other paraffin-embedded mammary glands sections were placed on silane-treated slides, and maintained at room temperature, as previously described [34]. Sections were incubated with the following antibodies: monoclonal antibody against VEGF SC-57496 (Santa Cruz Biotechnology, Santa Cruz, CA) at 1:100 dilution, monoclonal antibody against VEGFR-2 SC-6251 (Santa Cruz Biotechnology, Santa Cruz, CA) at 1:100 dilution, polyclonal antibody against metalloproteinase-9 (MMP-9) SC-6840 (Santa Cruz Biotechnology, Santa Cruz, CA) at 1:200 dilution and polyclonal antibody against COX-2 SC-1747 (Santa Cruz Biotechnology, Santa Cruz) at 1:100 dilution. Incubations were carried out overnight and then revealed using LSAB2 Kit HRP, rat (Dako-Cytomation, Carpinteria, CA) with diaminobenzidine (3,3′-diaminobenzidine tablets; Sigma, St. Louis, MO) as the chromogen and counterstained with hematoxylin. For each antibody, negative control slides consisted of sections incubated with antibody vehicle.

All tumors were examined by two blinded observers using a 400× magnification on light microscope (Nikon, Tokyo, Japan) connected to a digital camera (Coolpix 990; Nikon). From each specimen, ten fields of an immunostained section (VEGF, VEGFR-2, MMP-9 and COX-2) were chosen at random and captured with high-quality images (2048 × 1536 pixels buffer) and quantified using Image Pro Plus 4.5.1 (Media Cybernetics, Silver spring, MD). Histologic scores (H) for all immunomarkers were calculated using the formula H = ΣPi, where I is the intensity ranging from 0 (negative cells) to 3 (deeply staining cells) and P is the percentage of staining cells for each given i, with *P* values of 1, 2, 3, 4, and 5 indicating < 15%, 15–50%, 50–85%, > 85%, and 100% positive-staining cells, respectively, as previously described [28, 32]. These results were expressed as mean ± standard deviations.

### ELISA immunoassay

Peritoneal fluid was collected with 10 mL of PBS, pH 7.2 and centrifuged at 1500 rpm for 10 min. All serum samples were tested for VEGF, prostaglandin E_2_ (PGE_2_) and interleukin-10 (IL-10) antibodies using an enzyme immunoassay kit (Thermo Scientific, Waltham, MA, Cayman Chemical, Ann Arbor, MI and Thermo Scientific, Waltham, MA, respectively), as per the manufacturer’s instruction. These samples were performed on an automatic plate reader (Spectra Max; Molecular Devices, Sunnyvale, Calif) controlled by SoftMax software (Molecular Devices). The concentrations were calculated from standard curves and all samples were analyzed in triplicate.

### Flow cytometry

Peritoneal fluid was collected from each animal immediately after sacrificing, using a lavage of 10 mL of PBS at pH 7.2 for flow cytometry analysis (FACSCalibur, BD Biosciences, USA). The peritoneal cells were incubated with monoclonal antibodies PI anti-Mac-2 and FITC anti-F4/80 (Santa Cruz Biotechnology, Santa Cruz, CA) for 30 min each. Then, the cells were incubated with Fc blocking (clone 2.4G2) for 10 min. Ten thousand events were counted for each animal sample, and the analyses were performed in CellQuest (BD Biosciences, USA) and WinMDI 2.9 software packages.

### Statistical analysis

Data are expressed as mean ± standard deviations (SD). The statistical significance between açaí group and control was assessed using Student t-test, while the categorical data were expressed as percentages and evaluated by the Person Chi-square test (χ2). For VEGF, VEGFR-2, MMP-9 and COX-2 morphometric analysis, statistical calculations were carried out using the Stat-Xact-5 software program (CYTEL Software Corporation, Cambridge, MA). Survival curves were estimated by the Kaplan-Meier product-limit method using SPSS 13.0 software for Windows (SPSS Inc., Chicago, Illinois) and evaluated with the log-rank test. Significance for all statistical comparisons was set at *p* < 0.05.

## Results

### Breast tumor growth is inhibited by açaí treatment

**Fig. 1:**
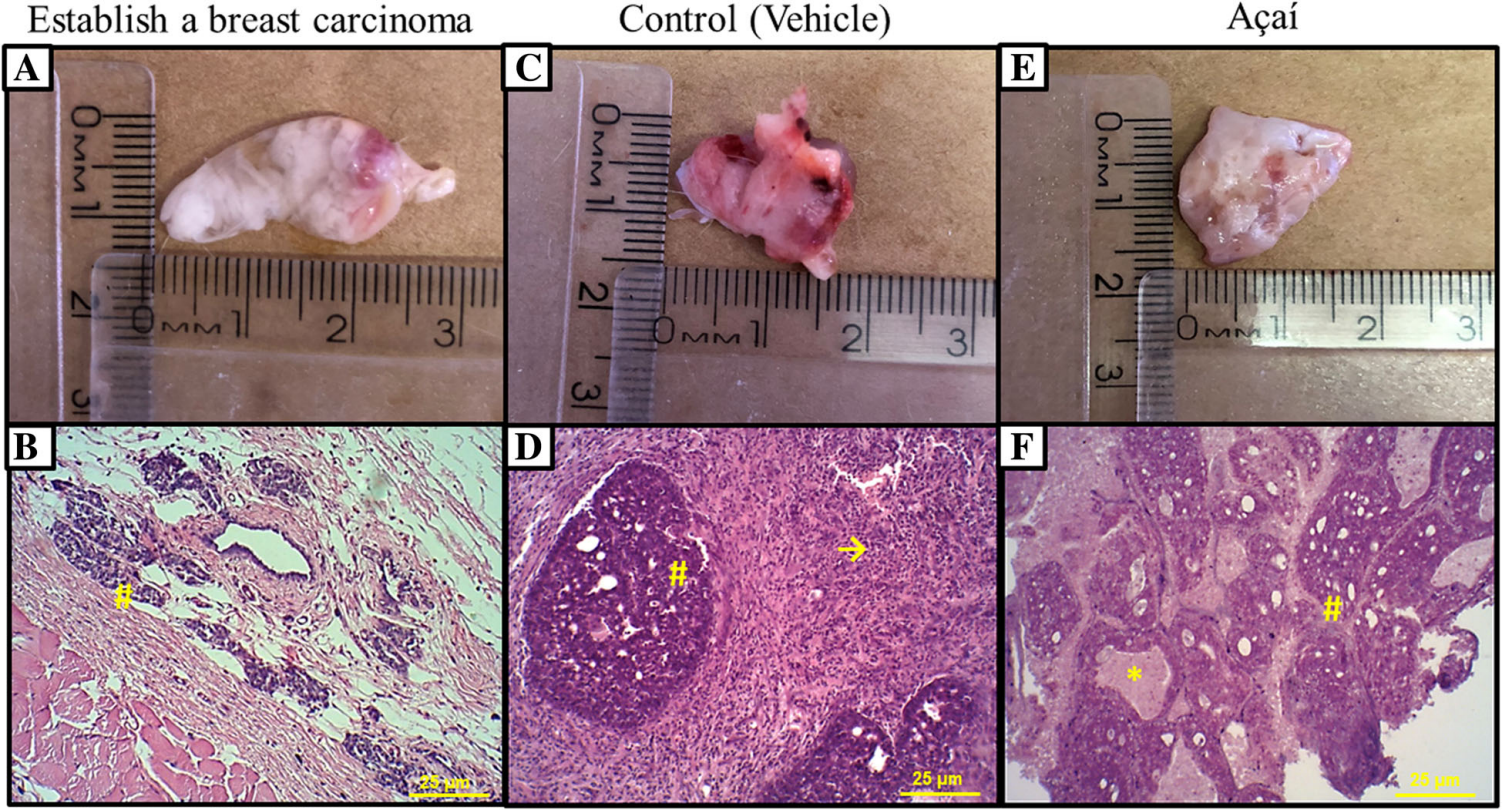
Establishment of the experimental model of breast cancer and treatment with açaí. (**a**) The morphological appearance of the mammary tumor in DMBA treated rats. (**b**) Histologically, observed the presence of area carcinoma invasion with hyperchromasia cells surrounding the fibrovascular stroma (#). DMBA induces breast cancer in control (**c**) and açaí (**e**) groups. (**d**) Area carcinoma invasion with hyperchromasia cells surrounding the fibrovascular stroma (#) and higher number of inflammatory cells (→). (**f**) We also observed invasive carcinoma (#), however, we noted a decrease of number of inflammatory cells (*)

**Fig. 2:**
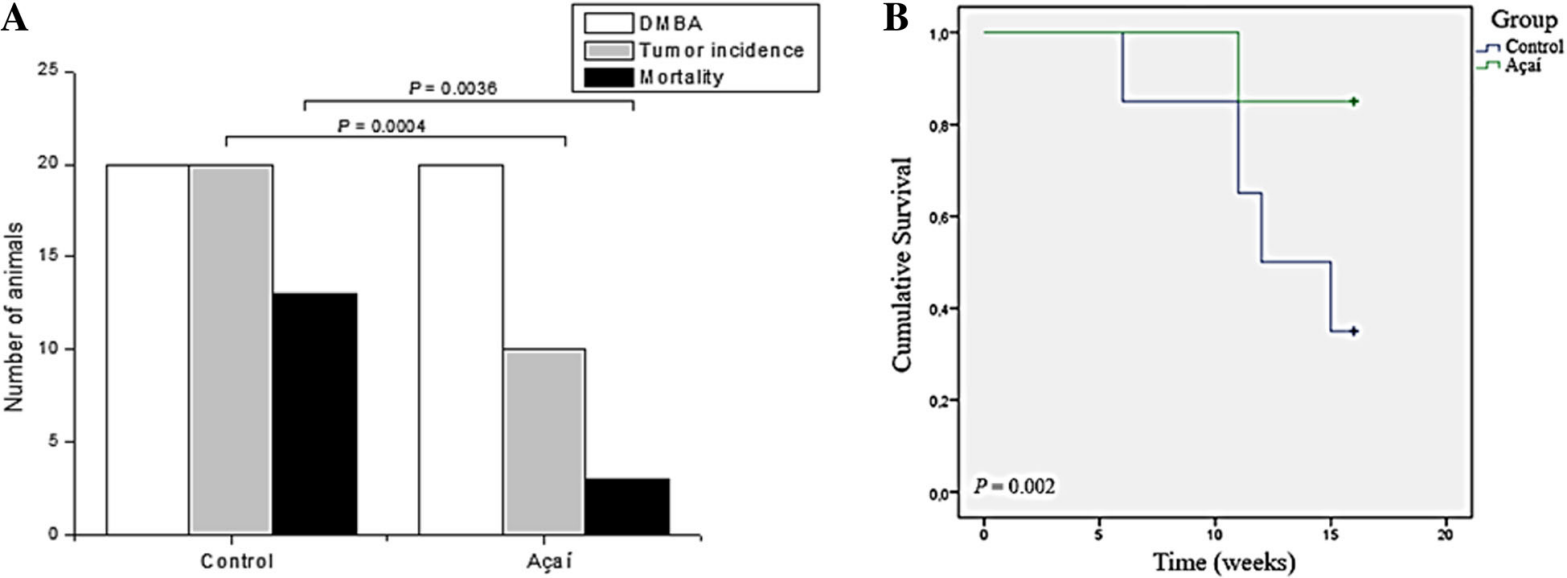
Tumor incidence and mortality with açaí treatment. The tumor incidence and mortality was statistically reduced in açaí group (**a**). Survival analysis indicates that açaí reduced the lethal effect of DMBA (**b**), control (blue line) and açaí (green line) groups

After 16 weeks of DMBA breast cancer treatment induction, the presence of the mammary carcinoma was confirmed by macroscopic and histological evaluation (Fig. 1a and b). The tumor mass was found in the mammary gland, where a characteristic cystic morphology associated with a well-developed vessels network could be observed, in addition to a markedly fibrotic pattern (Fig. 1a). Furthermore, the histological analysis reveals atypical clusters of hyperchromatic malignant cells separated by fibrovascular tissue (Fig. 1b). Moreover, along the establishment of the chemically induced breast cancer mice model, the tumor incidence and death rates were 93.2% and 43.8%, respectively. During the 16 weeks of establishment of mammary carcinoma mice model, 13 animals from the control group died (65% mortality rate). On the other hand, in the group treated with açaí extract, only 3 animals died (15% mortality rate). In the control group, the tumor incidence rate was 100%; however, in the group treated with açaí extract, the tumor incidence rate was markedly reduced to 50%. These data suggest that açaí extract could prevent the chemically carcinogenesis induced by DMBA-treatment (Fig. 2a). Furthermore, açaí treatment increased the overall survival when compared with control group (Fig. 2b); a cumulative survival of 15.15 weeks and 12.75 weeks was observed in the açaí treated group and in the control group, respectively (*P* = .002, long-rank test). In addition, in both groups, mammary tumors display adhesions and cystic pattern near the site of tumor induction (Fig. 1c and e); however, no significant difference in tumor volume (control: 4.151 ± 0.8 cm^3^; açaí: 3.971 ± 1.3 cm^3^) and weight (control: 3.012 ± 0.5 g; açaí: 2.52 ± 0.7 g) was noted. Finally, despite the fact that histopathological analysis did not reveal significant differences in the area of invasive carcinoma or in the number of hyperchromatic cells around fibrovascular stroma between the control group (Fig. 1d) and the açaí treated group (Fig. 1f), the presence of inflammatory clusters was markedly increased in the control group (Fig. 1d) when compared with the açaí treated group (Fig. 1f).

### Açaí extract diminishes the macrophage recruitment and release of inflammatory mediators in the breast tumor model

**Fig. 3:**
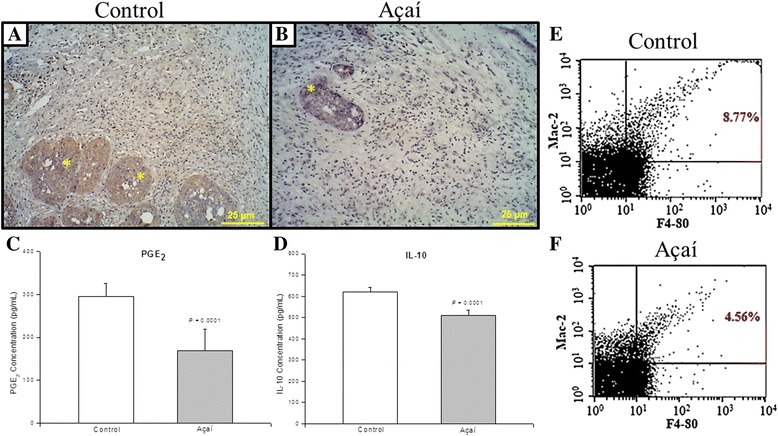
Anti-inflammatory effect of açaí on breast tumors. The immunoreactivity of COX-2 was detected predominantly in the glands (*) in the control (**a**) compared with açaí (**b**). PGE_2_ levels (**c**) and IL-10 (**d**) were higher in the control than in the açaí. FACS analysis (**e**, **f**) of the phenotype of macrophage (Mac-2/F4–80) revealed fewer macrophages levels in the treated açaí (**f**) than the control (**e**)

In order to investigate whether the reduced inflammatory infiltrate revealed by histological analysis in the açaí treated group could be attributed to the effects of açaí extract, we analyzed key mediators involved with inflammatory cascade, such as COX-2, PGE_2_ and IL-10. In addition, the presence of macrophage cells was also analyzed by immunostaining to F4–80/Mac-2. The immunohistochemical analysis revealed a high COX-2 staining in the breast tumor samples from the control group (Fig. 3a), while a weak staining pattern was observed in the breast tumor samples from the açaí treated group (Fig. 3b). Moreover, the histomorphometry analysis demonstrated that COX-2 level was significantly smaller (*P* = .0001) in breast tumors samples from açaí treated group (1.3 ± 0.5) when compared to the control group (41.1 ± 2.4). Lastly, in accordance with immunohistochemical data, the levels of PGE_2_, IL-10 and F4–80/Mac-2 positive macrophage cells were significantly smaller in the peritoneal fluid of the açaí treated group, compared with the levels found in the peritoneal fluid from the control group (Fig. 3c-f). Therefore, these results could reveal a potential anti-inflammatory activity of açaí extract.

### A decreased expression of angiogenic markers was induced by açaí treatment

**Fig. 4:**
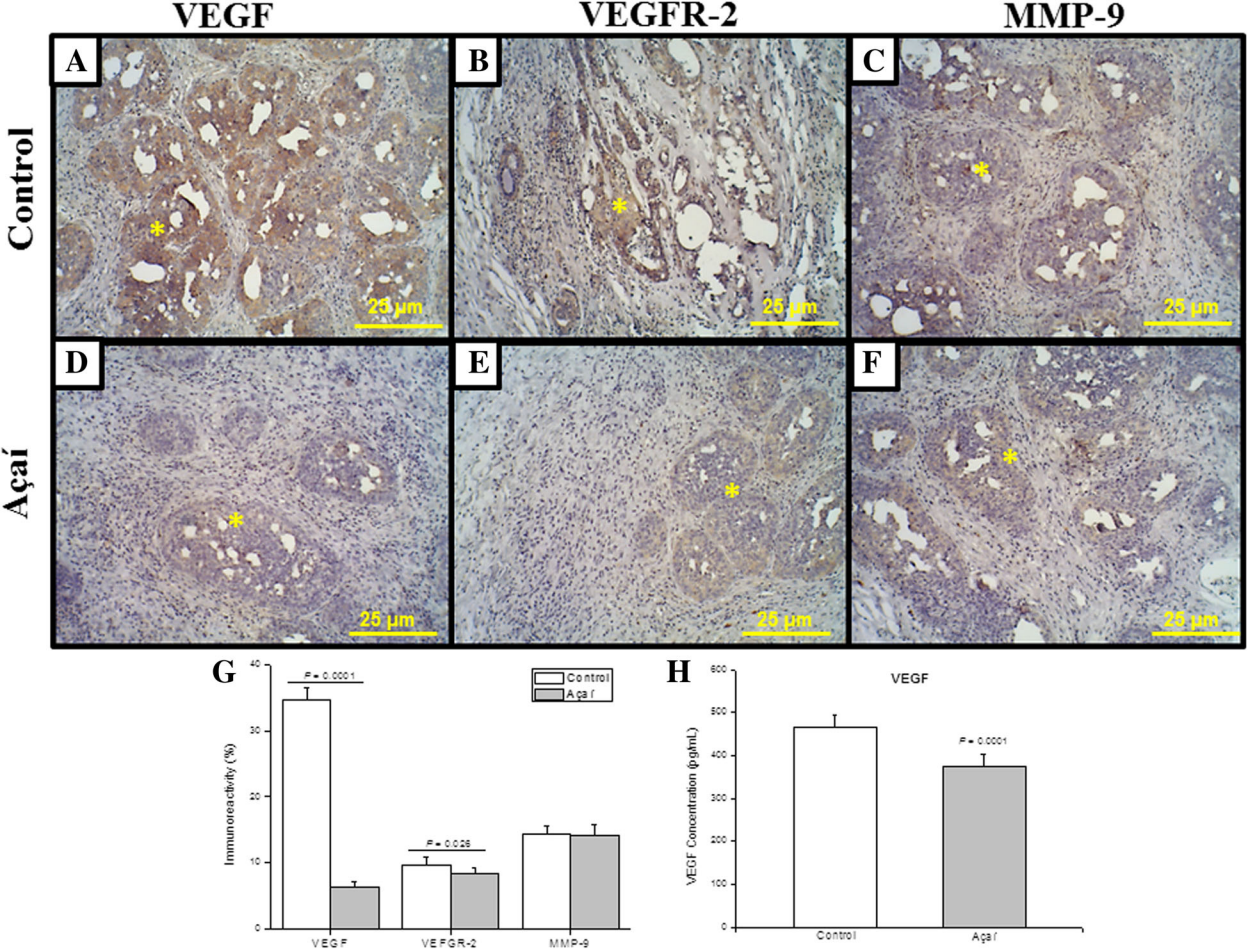
Açaí decreased angiogenesis markers immunodistribution in breast cancer. The immunoreactivity of VEGF and VEGFR-2 were detected predominantly around the glands (*) in control group (**a**, **b**). Treated breast cancer lesions (**d**, **e**) exhibited a significant decrease in reaction intensity (*). MMP-9 immunoreactivity was the same (*) in the control (**c**) and the açaí (**f**). Histomorphometry evaluations of VEGF, VEGFR-2 and MMP-9. ELISA analysis (h) indicated that the VEGF levels were higher in the control than in the açaí

The angiogenesis process is an essential step during tumor genesis and progression. In this sense, the protein expression of well-known pro-angiogenic factors was investigated. While no significative differences were observed in the levels of MMP-9 between control and açaí treated groups (Fig. 4c and f), the immunostaining of VEGF and its receptor VEGFR-2 reveals a substantial increase in the expression of these two proteins in the tumor stroma, as well as in the glandular compartment from control group samples (Fig. 4a and b). On the other hand, VEGF and VEGFR-2 were barely expressed in the tumor samples the from açaí treated group (Fig. 4d and e). Additionally, these data were corroborated by histomorphometry (Fig. 4g) and ELISA immunoassay analysis (Fig. 4h).

### Açaí extract exerts a protective effect on the DMBA-induced breast cancer model

**Fig. 5:**
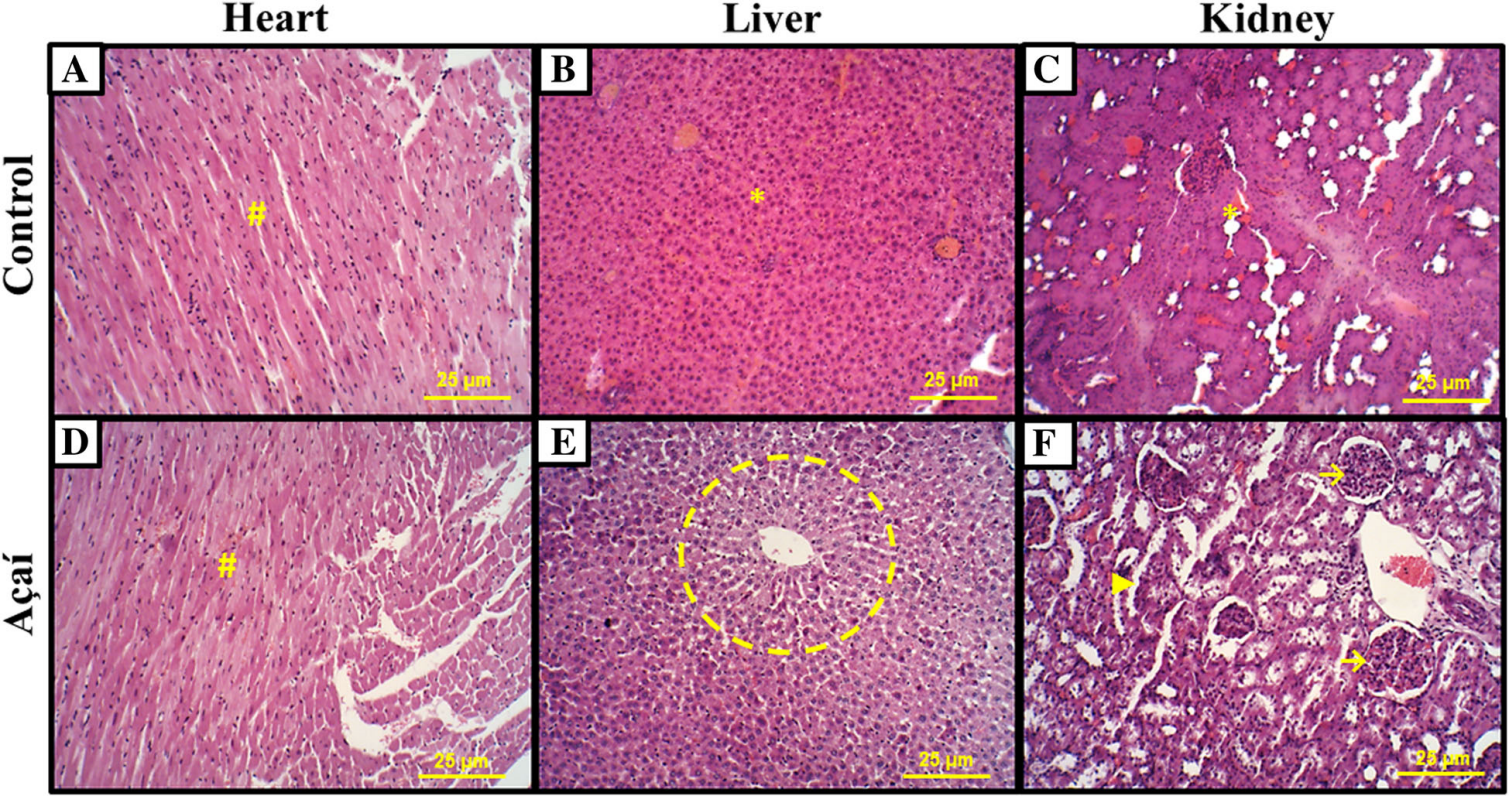
Açaí reduce toxicity effects in breast cancer experimental. Microscopic analysis: (**a**, **d**) no morphological difference in heart was observed with presence of cardiac muscle tissue well preserved (#). (**b**, **e**) In açaí treatment, we note the presence of centrilobular vein and cords of hepatocyte (circle) showed normal liver tissue. However, we observed higher toxicity effects in control liver with higher fibrosis, atypical cells and hemorrhagic microenvironment (*). (**c**, **f**) Similarly, in kidney analysis we also demonstrated increased toxicity in control (*), while in the açaí group, we observed the presence of numerous glomeruli (→) and apparent distal tubules (►)

No evidence of toxicity was noted in the açaí treatment based on food consumption, body weight and activity levels compared with controls. Heart histopathological revealed presence of cardiac muscle tissue preserved in both groups, with no morphological difference between the control group (Fig. 5a) and the açaí group (Fig. 5d). The histopathological results of the liver and kidney showed higher toxicity effects with increased fibrosis, atypical cells and hemorrhagic microenvironment in the control group (Fig. 5b and c). In the açaí treatment group, the histopathological of the liver revealed the presence of centrilobular veins and cords of hepatocyte with normal liver tissue (Fig. 5e), and the kidney showed the presence of numerous glomeruli and apparent distal tubules with preserved tissue (Fig. 5f). Raw data from tumor volume and weight, ELISA (PGE_2_, IL-10 and VEGF) and morphometric quantification (VEGF, VEGFR-2, MMP-9 and COX-2) are available in Additional file 1.

## Discussion

Breast cancer is a common neoplasm in women worldwide, second only to non-melanoma skin tumors, and has high rate of incidence and mortality [1, 2]. Nowadays, breast cancer treatments comprise surgery, chemotherapy, radiation, hormone and immunotherapy; however these therapies have side effects [17]. Moreover, the 5-year disease-free survival rate of stage II breast cancer is 75–90%, ~ 30% for stage III patients and 0–10% for stage IV breast cancer [35], highlighting the need for new treatment and prevention strategies. Natural products are being used and investigated for the discovery and development of new therapeutic antineoplastic compounds [36]. Previously, our group described the antitumorigenic action of açaí in a breast cancer cell line (MCF-7) due to açaí promoting a significant reduction in cell viability, changing cell morphological characteristics and inducing autophagy [26]. Corroborating this result, Freitas and colleagues [37] also recently revealed that açaí reduced cell viability of MCF-7 cell. However, these authors showed that the ethyl acetate fraction (EAF) is the most cytotoxic part of the hydroalcoholic extract of açaí seed and induced cell necrosis in the MCF-7 cells [37]. So, to our knowledge, this report is the first study to evaluate the effects of açaí against DMBA-induced mammary carcinogenesis in a rat model.

In the present study, açaí treatment decreased the number of deaths and the developed breast tumors experimental. We administered DMBA-induced rat mammary carcinoma with the cystic pattern, vascularized and fibrotic tissues. The tumor incidence and mortality rates of our model were similar to other studies with the same chemical inductor, dose and time; we found tumor incidences of 82% [38] and 100% [30] and survival rates of 40% after 17 weeks [39] or 60% after 20 weeks the induction [40], respectively. Although does not reducing the size of the tumor, our results indicate that açaí inhibits tumorigenesis effect of the chemical carcinogen DMBA and the incidence of breast experimental cancer, as previously described by Stoner et al. [41], using carcinogen N-nitrosomethylbenzylamine-induced tumorigenesis in the rat esophagus.

The inflammatory process is essential for the development and sustainability of the breast tumor mediated by the disbalance of the proinflammatory and anti-inflammatory cytokines [42]. To this end, we investigated COX-2, PGE_2_ and IL-10 signals to elucidate the action mechanisms of açaí in the breast cancer inflammatory process. COX-2 quickly results in the biosynthesis of prostaglandins, particularly PGE_2_ [43]. Elevated COX-2/PGE_2_ expression is associated with tumor aggressiveness such as bigger tumor size, positive lymph node metastasis of breast carcinoma and overall survival [14, 44]. We observed reduced COX-2, PGE_2_ and IL-10 levels in the açaí group, and these reductions were probably due to the high levels of anthocyanin [23] and polyphenols [45, 46] in the açaí. Several plant-derived compounds and natural products have been studied in breast tumors and showed reduction of COX-2 [47–51], PGE_2_ [48, 49] and IL-10 [49]. There are no studies demonstrating anti-inflammatory activity of açaí in in vivo breast cancer model, but we previously showed this effect in endometriosis experimental model, with reduction of COX-2, PGE_2_, nitric oxide levels and also reduced macrophages [28]. Our results suggest that açaí has an anti-inflammatory activity in breast experimental cancer.

Among immune cells recruited to the tumor site, macrophages are the most important and contribute to dysregulation of the pro-inflammatory cytokines. Interestingly, macrophages can exhibit high plasticity, play a pro-tumor role, stimulate angiogenesis, enhance tumor cell invasion, promote metastasis, survival, promote persistent growth, and are present at all stages of tumor progression [52–54]. Recently, Chen et al. [55] showed that tumor-recruit M2 macrophages could promote gastric and breast cancer metastasis by the M2-secreted chitinase 3-like protein 1 (CHI3L1) triggering mitogenic-activated protein kinase (MAPK) signaling pathway. These authors also described that the CHI3L1 has a potential to be a therapeutic target for metastatic cancer [55]. In our breast cancer experimental model, we observed a reduction of the macrophage phenotype in about 50% in the treated açaí group compared to the control. Therefore, this result suggests that acai can interfere with the macrophages survival pathways in breast cancer model.

As explained above, the activated macrophages can stimulate angiogenesis process. Several studies described that angiogenesis is important in the development of breast cancer, and overexpression VEGF has been associated with advanced stage, metastasis and relapse-free survival or overall survival in breast tumors [8–11, 56, 57]. In our study, açaí treatment reduced VEGF and VEGFR-2 expression. Similar results using açaí had previously been described in colon cancer cells [58]. In addition, the reduced VEGF expression was reported in breast cancer models treated with apigenin [59], luteolin [60], soy protein [61] and *Kalpaamruthaa* [51], as well as a decrease in VEGFR-2 expression treated with apigenin [59] and melatonin [62]. Interesting, several authors reinforce the importance of VEGF in mammary carcinogenesis and suggest that this factor can contribute to a new classification of breast cancer subtypes [10, 63, 64]. So, the inhibition of this pathway might be the target of therapies against breast cancer.

Açaí treatment did not show any signs of toxicity on the animals according to their food consumption, body weight and activity levels compared with controls, as was previously described in colon [65] and in urinary bladder carcinogenesis [66]. Furthermore, in the present study, no cytotoxicity was observed in the liver and kidney in the groups that received açaí treatment compared to negative controls. In this way, Ribeiro et al. [29] investigated the genotoxicity of açaí in bone marrow, peripheral blood, liver and kidney cells of mice, and demonstrated that gavage administration of açaí was not genotoxic in these animals, and açaí’s components may be exploited as a promoter of good health [29]. Similarly, Marques et al. [67] showed that açaí had no significant genotoxic effects in the leukocytes, liver, bone marrow and testicular cells. These results demonstrate that açaí is a safe and functional food ingredient for cancer chemoprevention studies [66].

**Fig. 6:**
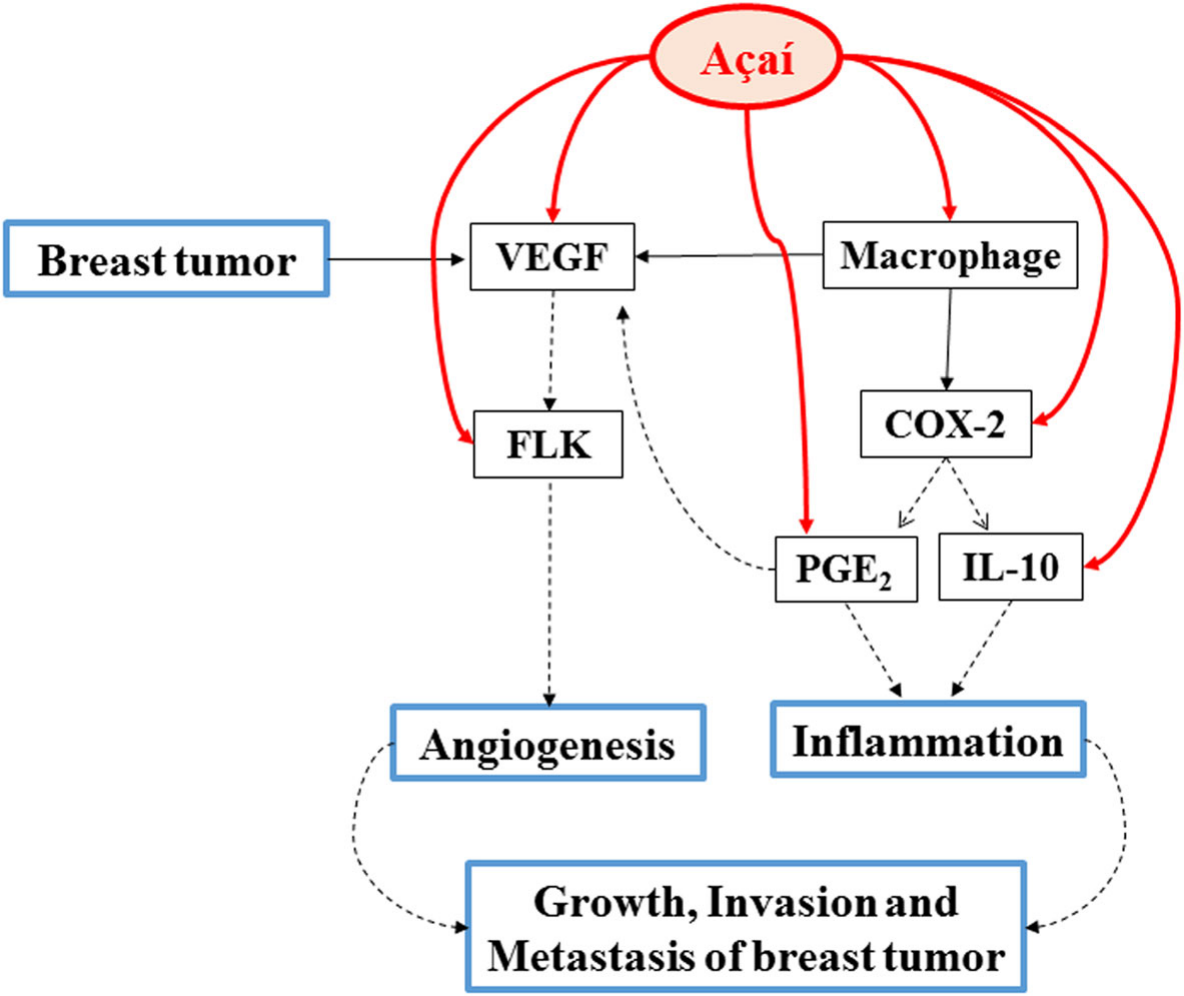
Proposed mechanism of açaí in breast cancer. In the breast tumor microenvironment, the macrophages are essential promoting the angiogenesis process and inflammation because they lead to increases in the VEGF, VEGFR-2, COX-2 and PGE2 levels. Açaí acts in this pathway and reduces the number of activated macrophages resulting in the decrease of the signaling pathways and the levels of these genes, suppressing growth or reducing the size of breast tumor

Finally, based on the results of this study and the previous ones, we proposed a mechanism for the therapeutic effects of açaí in the breast cancer development (Fig. 6). Macrophage plasticity plays a key role in inflammation and angiogenesis in the mammary carcinoma, acting as an important source of VEGF in the establishment and growth of breast tumor [68–71]. High concentrations of VEGF produced both by the activated macrophages and breast cancer cells returns to bind with VEGFR-2 and stimulated angiogenesis in the tumor microenvironment. Furthermore, the polarization of macrophages induced COX-2/PGE_2_ signals enhancing the inflammatory process and stimulated the expression of VEGF [72]. On the other hand, açaí decreased the number of activated macrophages on the tumors resulting in downregulating the VEGF/VEGFR-2 signals and in the PGE_2_ levels suppressing the inflammation, angiogenesis and growth of breast tumor.

## Conclusions

In conclusion, we demonstrated that açaí exhibits antitumorigenic activity in DMBA-induced breast cancer mainly by its antiangiogenic and anti-inflammatory effects. Açaí may modulate the progress of breast cancer by decreasing the presence of the activated macrophages in the tumors, which leads to reductions of VEGF, VEGFR-2, COX-2, PGE_2_ and IL-10 levels, thereby supporting the use of açaí for an adjuvant treatment together with chemotherapy drugs. The mechanisms of açaí are not completely understood and we will continue to study this extract.

## Additional file

Additional file 1: Raw Data. Raw data from tumor volume and weight, ELISA (PGE2, IL-10 and VEGF) and morphometric quantification (VEGF, VEGFR-2, MMP-9 and COX-2). (XLSX 37 kb)

## Abbreviations

CEA: Animal Care and Use Committee
CHI3L1: Chitinase 3-like protein 1
COX-2: Cyclooxygenase 2
DMBA: 7,12-dimethylbenzanthracene
EAF: Ethyl acetate fraction
H: Histologic scores
HE: Hematoxylin-eosin
IL-10: Interleukin-10
MAPK: Mitogenic-activated protein kinase
MMP-9: Matrix metalloproteinase-9
PBS: Phosphatebuffer saline
PGE_2_: Prostaglandin E_2_
SD: Standard deviations
UEZO: University State of West Zone
VEGF: Vascular Endothelial Growth Factor
VEGFR-2: Vascular endothelial growth factor receptor 2
χ^2^: Chi-square test of Person
